# The *How* and *Why* of Consciousness?

**DOI:** 10.3389/fpsyg.2018.02173

**Published:** 2018-11-21

**Authors:** Tim S. Meese

**Affiliations:** Aston Laboratory for Immersive Virtual Environments, School of Life and Health Sciences, Aston University, Birmingham, United Kingdom

**Keywords:** consciousness, free will, social acceptability, personal choice, qualia, epiphenomenalism, physicalism, philosophy of mind

Understanding how subjective experience can arise from the nuts and bolts of matter is known as the hard problem of consciousness (Chalmers, 1996). Nobody has come close to solving this. One approach, type-A materialism (Chalmers, 2002) (hereafter, *hard-core physicalism*), simply dismisses the hard problem altogether. On this view, nothing about subjectivity or qualia needs explanation beyond their functional underpinnings: consciousness is an illusion, and the states of our inner world, merely dispositions to act (Churchland, 1985; Dennett, 1988). Should we hope that by studying the “illusion” of consciousness (Dennett, 2003) we might unpick the real mechanism, in the way, for example, psychologists understand motion perception by studying the waterfall illusion (Mather et al., 2008)? According to hard-core physicalists—no, it's illusions all the way down; it has to be, because there is no true mechanism of consciousness to be revealed, it is simply the name we give to the inner state of the complex machine we are: the lights are not really on, it only seems that way.

Perhaps hard-core proponents are safer with the less controversial statement that consciousness is not what it seems. Numerous examples from experimental psychology support this: contrary to daily experience, our sensations and/or perceptions of the world are inhomogeneous (Baldwin et al., 2012), internally constructed (Ramachandran and Gregory, 1991), lossy (Pashler, 1988), and not even needed for some behaviors (Weiskrantz, 1985). However, I do not take these observations to empower the central hard-core claim that while direct experience is undeniably felt it must be discredited if we are to understand what needs to be understood about consciousness (Dennett, 2001). Indeed, this position leaves some people feeling as empty as the explanation itself (e.g., see Nagel, 2017). Might there be another answer, one that preserves the third-person tradition of objective science, while acknowledging the importance of there being *something it is like* (Nagel, 1974; Jackson, 1982) to be conscious?

Type-B materialism (Chalmers, 2002) (hereafter, *soft-core physicalism*) is a widespread alternative. This position is common in neuroscience, where the hunt is on for the neural correlates of consciousness: the neural states that identify with conscious experiences. However, because identity is not explanatory, soft-core physicalism ends up looking more like property dualism than materialism (Chalmers, 1997).

## Is there another way?

Oakley and Halligan (2017) (hereafter O&H) believe so. They understand that consciousness is not a control mechanism for our behavior, but a passive observer of our life narrative, a narrative that emerges from competition between the challenges and demands of the unconscious. (O&H prefer the term non-conscious; I draw no distinction.) We have no free will (e.g., Harris, 2012; Miles, 2015), this much is clear under materialism, compatibilist claims to the contrary (Dennett, 1984) being wordplay that shifts the meaning of the term *free will*: I am not being coerced into writing this article, I do it of *my own free will*; not just a figure of speech but, for example, the basis of a framework for our justice system, one we need, but one that reinforces the delusion^1^. For some, this delusion is a good thing (e.g., Smilansky, 2002), the concern being that the public might not accommodate the knowledge of delusional agency for the better (see *The truly nefarious neurosurgeon* in Dennett, 2013). However, recent experimental work suggests the opposite conclusion: that such beliefs can induce prosocial behavior (Casper et al., 2017). Another perspective steps outside the philosophical debate (Lavazza, 2016). Whilst acknowledging the attendant legal and moral problems, Lavazza suggests tests of cognitive control from which an index of an agent's operational capacity for a pragmatic form of free will could be derived.

In sum, the compatibilist view is that sentient biological units have the elbow room (Dennett, 1984) to operate free of coercion, but the purist (Harris, 2012) always finds a causal chain of events leading to the current disposition—there is no freedom to be found (Harris and Dennett, 2016).

O&H build on this disconnection between conscious experience (of will) and execution of action, suggesting consciousness is merely a side-effect of something else going on; an epiphenomenon, like the colors of the rainbow. They suggest it is the internal broadcasting—a delightful concept—of a selective personal narrative that defines the wick of our life during its transfer to memory. That we are merely subjects of unconscious authoring is certainly plausible (Nisbett and Wilson, 1977; Libet, 1985; see Bayne, 2011 for criticism) and, for some, an intuitive account of our reality and our self (Harris, 2012; Miles, 2015).

There are two main problems with O&H's thesis on consciousness. The first is common to all accounts that appeal to epiphenomenalism: the simple fact is, we can *talk* about consciousness. This is not trivial; it means the thing we call consciousness can influence the underlying system (by causing it to speak), and in philosophy of mind, epiphenomena do not have causal feedback (e.g., Megill, 2013), so consciousness cannot be epiphenomenal (Blackmore, 2004; Bailey, 2006; Robinson, 2015). For a defense of epiphenomenalism to work, it would have to be that when I talk of consciousness, I'm using that word to refer to something else: the mechanistic underpinnings. But this is not how it feels, when I talk to you about consciousness, I believe we are both referring to the same felt sense that the lights are on. Borrowing from Bailey (2006), if the proposed epiphenomenal status of consciousness seems counter-intuitive (O&H), the original intuition that is being countered cannot have been derived from knowledge of consciousness. If it was not clear before, perhaps we begin to see why the elimination of qualia and a first-person perspective through hard-core physicalism is so attractive, if wildly counter-intuitive (Churchland, 1985).

The second problem is that O&H's theory does not even *require* consciousness—we can envisage a machine that is programmed to store only some of its internal operations in memory, and call that a personal narrative, but it does not follow that this will imbue the machine with consciousness. Others have made similar slips. Humphrey (1986) developed a strong case that we are social creatures and need to understand ourselves to understand others; the feedback loop in this self-reflection is the origin of consciousness. It's a nice idea, and such a loop may have value, but a simulation of servo control would include a feedback loop without needing to be conscious; making the system social does not change that.

The reason O&H (and others) end up with something plausible but not persuasive is that their starting point is wrong. There is a tendency for workers on consciousness to look toward information processing, or brain neurophysiology, or human needs and behavior, to roll out something that—ba-ba-boom—gives us consciousness (this is typical of soft-core physicalism). Not only does the explanatory gap remain (Levine, 1983) but the case that demands the emergence of consciousness qua consciousness is not made either. So is there a better starting point?

I think there is. Instead of arguing about whether there is a hard question of consciousness (i.e., whether there is a phenomenon that needs explaining; Chalmers, 1997), for which no solution is in sight, it might be better to ask, what do we have that *requires* consciousness (e.g., Humphrey, 2006)?

To illustrate the *why* (and not worry about the *how*), I consider two possible answers to the question above^2^. The first is qualia, the individual instances (over space and time) of our subjective conscious experiences. By definition, qualia *require* consciousness. Putting aside the possibility they are not what they seem (Dennett, 1988), might we need them (in some sense), and hence became conscious? How are qualia used? When we see “red,” for example, we are (typically) experiencing our belief (derived from post-receptoral computations in the brain) about the spectral reflectance properties of a surface we are observing (even if we lack the technical knowhow to express it that way^3^). When we *say* “I see ‘red”' we are using the symbols of language to broadcast that belief externally. Like the word “red,” the quale “red” is not a property of the outside world, but also a symbol (or tag), this time in the domain of consciousness. It is an *internal* broadcast of our belief (typically with greater precision than word symbols) about the external world. (Other qualia do this for other sensory modalities, and for internally generated signals too). Qualia are valuable. However, appealing to the symbolic nature of qualia as a justification for being conscious puts us on shaky ground: symbols are valuable for information processing whether the system is presumed conscious or not (Marr, 1982).

The second answer is perhaps more promising: we have the delusion of free will^4^. This operates on our internal models of: the world stage, the players, our self and our feelings—our qualia. Consciousness is a necessary vehicle for this delusion, and, by association, a colorful source of internal virtual lighting. For me to experience myself, as though in the driving seat, as though I have transcended my neurons (even if I hold a scientific belief I do not), I must be conscious^5^. Our question thus becomes: what is the evolutionary benefit in having the delusion of free will?

I think we find hints of what might be the answer in both O&H and Humphrey. My operating system/reporting mechanism is good, but imperfect; to tolerate this deficiency in myself and others, I can attribute my perceptions of shortcomings, idiosyncrasies, and inconsistencies to personal choice. This is executed by sharing a personal narrative through external broadcast (O&H), and constructing a model of the other (Humphrey, 1986) with deviations from myself as a starting point. This sanctions the likes and dislikes held by others with which I might not agree but which (in my tribe at least) I can tolerate since, believing they derive from personal authorship—something I (laughably) value in myself—, I am excused any destructive inclination I might have for conflicting (and potentially deficient) biological hardware that is sharing my space. I trust the other can do likewise by a similar process. A social alliance, then; one that circumvents a needless invocation of survival of the fittest. In a nutshell: the delusion of free will demands consciousness and engenders excuse (of others, but self too); it smooths over the cracks, most of the time. This is the basis of social living, from which our species has most surely benefitted. This is not to say that cooperation and altruism need the free will delusion to emerge (e.g., Santos et al., 2008) but it seems likely it would help.

I applaud O&H for highlighting that our powers of control are not driven by consciousness, but they have not solved (or even tackled) the hard problem, and neither have I. Rainbows are not illusions, and even if they were, we would still need to understand their realization by the brain. But I have suggested a reason why the rainbow of our mind exists: we need consciousness to express the delusion of free will.

It is ironic then, that by unpicking the delusional nature of free will (Harris, 2012), a delusion from which we benefit, we become better placed to understand the wrong-doing of others, enriching our society with compassion, given we know they are conscious too.

## Author contributions

The author confirms being the sole contributor of this work and has approved it for publication.

### Conflict of interest statement

The author declares that the research was conducted in the absence of any commercial or financial relationships that could be construed as a potential conflict of interest.

## Footnotes

^1^It is worth emphasizing the distinction between illusion and delusion. The first is representational and pertains to the senses, the second is conceptual and pertains to higher-level beliefs. The dividing line is probably murky.

^2^A third possibility is offered by Cashmore (2010), who points out that our sense of agency also brings the burden of responsibility; a pro-social factor.

^3^Which demonstrates the power of direct experience over language.

^4^We can imagine being conscious without the delusion of free will, but we cannot imagine having the delusion of free will without being conscious.

^5^This might serve as a working definition of consciousness: if a system has the Gestalt expression that it is something more than the sum of its parts, it is conscious.

## References

[B1] Bailey A. R. (2006). Zombies, epiphenomenalism, and physicalist theories of consciousness. Can. J. Philos. 36, 481–510. 10.1353/cjp.2007.0000

[B2] Baldwin A. S. Meese T. S. Baker D. H. (2012). The attenuation surface for contrast sensitivity has the form of a witch's hat within the central visual field. J. Vision 12, 1–17. 10.1167/12.11.23 23104816

[B3] Bayne T. (2011). Libet and the case for free will skepticism, in Free Will and Modern Science, ed Swinburne R. (Oxford: Oxford University Press), 25–46.

[B4] Blackmore S. (2004). Consciousness: An Introduction, 2nd Edn Oxford: Oxford University Press.

[B5] Cashmore A. R. (2010). The Lucretian swerve: the biological basis of human behaviour and criminal justice system. Proc. Natl. Acad. Sci. U.S.A. 107, 4499–4505. 10.1073/pnas.0915161107 20142481 PMC2842067

[B6] Casper E. A. Vuillaume L. Magalhaes De Saldanha da Gama P. A. Cleeremans A. (2017). The influence of (dis)belief in free will on immoral behaviour. Front. Pyschol. 8:20 10.3389/fpsyg.2017.00020 PMC5239816 28144228

[B7] Chalmers D. J. (1996). The Conscious Mind: In Search of a Fundamental Theory. Oxford: Oxford University Press.

[B8] Chalmers D. J. (1997). Moving forward on the problem of consciousness. J. Conscious. Stud. 4, 3–46.

[B9] Chalmers D. J. (2002). Consciousness and its place in nature, in Blackwell Guide to the Philosophy of Mind, eds Stich S. Warfield T. (Oxford: Blackwell), 102–142.

[B10] Churchland P. M. (1985). Reduction, qualia, and the direct introspection of brain states. J. Philos. 82, 8–28. 10.2307/2026509

[B11] Dennett D. C. (1984). Elbow Room: The Varieties of Free Will Worth Wanting. Oxford: Oxford University Press.

[B12] Dennett D. C. (1988). Quining qualia, in Consciousness in Contemporary Science, eds Marcel A. Bisiach E. (Oxford: Oxford University Press), 381–414.

[B13] Dennett D. C. (2001). The Fantasy of First-Person Science. Available online at: https://ase.tufts.edu/cogstud/dennett/papers/chalmersdeb3dft.htm

[B14] Dennett D. C. (2003). The Illusion of Consciousness. TED Talk. Available online at: https://www.ted.com/talks/dan_dennett_on_our_consciousness

[B15] Dennett D. C. (2013). Intuition Pumps and Other Tools for Thinking. London: W. W. Norton & Company Ltd.

[B16] Harris S. (2012). Free Will. New York, NY: Free Press.

[B17] Harris S. Dennett D. C. (2016). Free Will Revisited. Available online at: https://samharris.org/podcasts/free-will-revisited/

[B18] Humphrey N. (1986). The Inner Eye. London: Faber and Faber.

[B19] Humphrey N. (2006). Seeing Red: A Study in Consciousness. London: Harvard University Press.

[B20] Jackson F. (1982). Epiphenomenal qualia. Philos. Q. 32, 127–136. 10.2307/2960077

[B21] Lavazza A. (2016). Free will and neuroscience: From explaining freedom away to new ways of operationalizing and measuring it. Front. Hum. Neurosci. 10:262. 10.3389/fnhum.2016.00262 27313524 PMC4887467

[B22] Levine J. (1983). Materialism and qualia: the explanatory gap. Pac. Philos. Q. 64, 354–361. 10.1111/j.1468-0114.1983.tb00207.x

[B23] Libet B. (1985). Unconscious cerebral initiative and the role of conscious will in voluntary action. Behav. Brain Sci. 8, 529–539. 10.1017/S0140525X00044903

[B24] Marr D. (1982). Vision. London: MIT Press.

[B25] Mather G. Pavan A. Campana G. Casco C. (2008). The motion after-effect reloaded. Trends Cogn. Sci. 12, 481–487. 10.1016/j.tics.2008.09.002 18951829 PMC3087115

[B26] Megill J. (2013). An argument against epiphenomenalism. Eur. J. Anal. Philos. 9, 5–17.

[B27] Miles J. B. (2015). The Free Will Delusion. Kibworth: Matador.

[B28] Nagel T. (1974). What is it like to be a bat? Philos. Rev. 83, 435–450.

[B29] Nagel T. (2017). Is consciousness an illusion? Review of: D. C. Dennett (2017) From bacteria to Bach and back: The evolution of minds, in The New York Review of Books, Vol. 64, eds Bowen E. Winslow-Yost G. Crowther P. Just J. Katzenstein A. (London: W. W. Norton & Company Ltd), 4.

[B30] Nisbett R. E. Wilson T. D. (1977). Telling more than we can know: verbal reports on mental processes. Psychol. Rev. 84, 231–259. 10.1037/0033-295X.84.3.231

[B31] Oakley D. A. Halligan P. W. (2017). Chasing the rainbow: the non-conscious nature of being. Front. Psychol. 8:1924. 10.3389/fpsyg.2017.01924. 29184516 PMC5694471

[B32] Pashler H. (1988). Familiarity and visual change detection. Percept. Psychophys. 44, 369–378. 10.3758/BF03210419 3226885

[B33] Ramachandran V. S. Gregory R. L. (1991). Perceptual filling in of artificially induced scotoma in human vision. Nature 350, 699–702. 10.1038/350699a0 2023631

[B34] Robinson W. (2015). Epiphenomenalism, in The Stanford Encyclopedie of Philosophy. Ed Zalta E. N. Available online at: https://plato.stanford.edu/entries/epiphenomenalism/

[B35] Santos F. C. Santos M. D. Pacheco M. (2008). Social diversity promotes the emergence of cooperation in public goods games. Nature 454, 213–216. 10.1038/nature06940 18615084

[B36] Smilansky S. (2002). Free will, and fundamental dualism, and the centrality of illusion, in The Oxford Handbook of Free Will, ed Kane R. (Oxford: Oxford University Press), 489–505.

[B37] Weiskrantz L. (1985). Blindsight: A Case Study and Implications. Oxford: OUP.

